# Why Bad Boys Always Get the Girl and Other Tales of Evolutionary Madness

**DOI:** 10.1371/journal.pbio.0030057

**Published:** 2005-01-25

Biologists studying the evolution of mate choice have their work cut out for them. Not only is there little agreement on how best to determine the interplay between mate choice and fitness, there's not even consensus on how to estimate fitness. (Fitness being an individual's success in passing their genes on to future generations.) Why females fall for captivating males that give them nothing but trouble is especially puzzling. Such males tend to contribute their genes and little else, leaving the female to spend precious resources bearing and raising her young, efforts that often cut her life short. Of course, females are not all innocence and light in the mating game: some black widow species, for example, famously make dad a post-coitus snack. Still, females typically incur more costs than males in rearing offspring, especially when they choose flashy mates. So why do they do it?

One model holds that females put up with deadbeat dads because the benefits, though indirect, outweigh the costs: that is, attractive males are more likely to grace their offspring with good genes that increase survival (“I'm so fit I can afford to waste energy on this excessively exuberant tail”) or with sexy traits that confer mating success (“my magnificent plumage may decrease my survival, but I get lots of dates”). Another model argues that selection for such indirect benefits is much weaker than direct selection on genes that affect mate preference and thus is likely to exert little influence on mating preference.

In a new study, Megan Head and her colleagues navigate this intellectual minefield by studying the mating behavior of crickets. The authors paired females with either “attractive” or “unattractive” males (see below) and measured a variety of fitness components to estimate the overall fitness consequences of the various unions. Female crickets, they found, pay a high price for mating with attractive males. But when the fitness consequences for their sons and daughters are taken into account, mate costs are balanced by, and may even be outweighed by, the indirect benefits of spawning offspring with elevated fitness. This benefit stems in large part, the authors argue, from siring sexy sons.

How does one distinguish lothario from loser in the cricket world? By running a cricket tournament, of course. For crickets to mate successfully, the female must mount the male so their genitalia align. Noting that females produce more eggs for males they mount quickly, the authors use time to mount as a measure of male attractiveness.[Fig pbio-0030057-g001]

**Figure pbio-0030057-g001:**
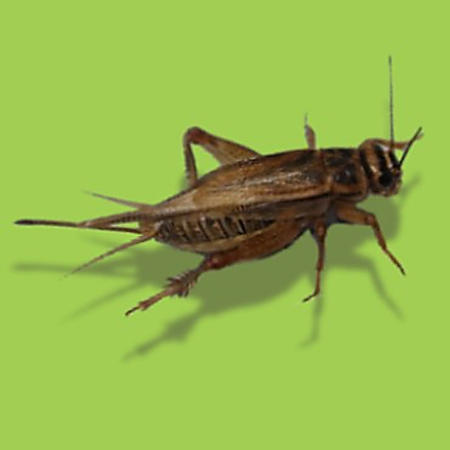
An attractive cricket?

In the first round of the tournament, Head and colleagues paired males with a randomly assigned female; after mounting, but before copulation, the couples were separated. This continued until half of all females had mounted a male. (Under tournament rules, crickets had to be in the first half of a given category to qualify for the next round.) In round two, a new female was randomly assigned to each male. Males that had been mounted in the first round and remounted in the second were deemed “attractive.” Males rebuffed in the first round that remained unmounted longest in round two were “unattractive.” Females were randomly assigned to males that were either attractive or unattractive. Equivalent males were swapped out every seven days to control for any individual quirks that might bias the results.

To estimate the total fitness of the participants, the authors measured both direct and indirect fitness components, such as female hatching success and reproductive effort (egg number and size), as well as sons' attractiveness and the number of eggs laid by daughters. Females that mated with attractive males produced daughters that laid more eggs within a given time and sons that were more attractive, though they had lower survival. Thus, by evaluating both the direct effects of female lifetime fecundity and the indirect effects of offspring fitness, the authors determined the net consequences of a mating strategy. And once again, it's mom's sacrifices that keep things on track.

With this approach, Head and colleagues bridge the gap between empirical studies of mating choice evolution, which rely largely on rate-insensitive measures (such as counting grandchildren), and theoretical studies, which typically use rate-sensitive measures. Their results suggest that there may be selection for choosing costly mates and that generating a reliable analysis of the fitness consequences requires a long view: look at the reproductive success of mom's sons and daughters before judging her bad taste in mates.

